# Midwifery

**Published:** 1836-07-01

**Authors:** 


A PRACTICAL TREATISE ON MIDWIFERY. By *Robert Collins*, M.D. Octavo, pp. 626. London, 1835.

PRACTICAL OBSERVATIONS ON VARIOUS SUBJECTS RELATING TO MIDWIFERY. By *James Hamilton*, M.D. F.R.S.E. Octavo, pp. 317. Edinburgh, 1836.

OBSERVATIONS ON THE PROPRIETY OF INDUCING PREMATURE LABOUR IN PREGNANCY, COMPLICATED WITH TUMOR. By Dr. *Ashwell*.

[Guy's Hospital Reports.]

IT has rarely fallen to our lot, as medical reviewers, to meet with a work which is so full of sound practical instruction, and, at the same time, so guiltless of unnecessary amplification, as this Treatise by Dr. Collins. It contains a faithful record of what the author witnessed and what he did during the seven years that he was attached to the Dublin Lying-in Hospital—certainly one of the noblest and best-managed charitable institutions of our land—as chief physician or master. During that period 16,414 women gave birth to 16,654 children. The particulars of each case, in reference to the age of the patients, the duration and character of the labour, the occurrence or not of casualties, the sex of the infant, and so forth, were carefully noted; and the immense mass of authentic data thus accumulated has been faithfully grouped and arranged under different sections by the enlightened author. There is no theory, or speculative reasonings admitted into the work. It is a simple registry of well-attested occurrences, prefixed or appended to which are the more important deductions which naturally flow from the premises, and a few practical instructions, all of which bear the stamp of a most cautious and diligent observation. If the precepts and practice of such a physician as Dr. Collins were more generally known and followed, we are confident that not only many a woman might be spared, in the most trying time of Nature's suffering, hours and days of pain and misery, but that scores of valuable lives might be saved. The kind and affectionate regard for the welfare of his patients which Dr. Collins expresses on all occasions is as creditable to his feelings as his admirable instructions are to the excellence of his judgment.

And certainly no department of our profession requires so much the union of a good heart and a clever head as that of midwifery.

Like every experienced practitioner, Dr. Collins deprecates, above all things, any hurry or premature interference in the management of labours, and he is right in tracing many of the distressing accidents, to which consulting accoucheurs are so frequently called, to the rashness and meddling bustle of the young and over-officious. His motto may be truly said to be "nec temere, nec timide." One leading object of his work is to inculcate the extreme danger to the patient, and the mortifying distress to the accoucheur himself of a too hasty adoption of manual or instrumental assistance in tedious and difficult labours. Some may possibly be of opinion that our author is rather too much disposed to sacrifice the life of the infant to the welfare of the parent, and we are ready to admit that sometimes he recommends the employment of the perforator and crotchet, when other physicians might be inclined to use the forceps or attempt to turn the child. Independently, however, of the perfect justifiableness and propriety of the principle alluded to, (and we do not hesitate to avow that as for ourselves we should always act upon it,) it may be very fairly presumed that small indeed is the number of children saved by the opposite course of treatment, such as is pursued on the Continent where the mortality among parturient women is well known to be so much greater than in this country. Numerous are the cases, as we shall shew in the sequel, where the safety of the mother has been most grievously endangered for the purpose of saving her offspring, and, after all, the child when born has been found not only *dead*, but absolutely *putrid*. Such a mistake may be generally avoided in the present day, at least by an accoucheur who has made himself practically acquainted with obstetrical auscultation. On many occasions the practice must depend on the results of an accurate stethoscopic examination. If the child is ascertained to be dead, the physician need have no scruple in using the perforator, whenever not to say the absolute safety, but the welfare and comfort only of the parent imperatively require a speedy termination of the labour. Dr. Collins makes allusion to the important treatise on obstetrical auscultation, published a few years ago by his former assistant, and present successor Dr. Evory Kennedy. We have reason to believe that the copious analytic review which we gave of that work two years ago, has greatly contributed to a widely diffused knowledge of its important contents.

In the following pages we propose to lay before our readers a condensed and accurate summary of the leading facts and doctrines contained in Dr. Collins' Practical Treatise.

We trust, however, that this notice will not supersede a direct appeal to the work itself. It ought to be perused, or rather studied by every medical man, who engages in the practice of midwifery, and to the public lecturer, the copious and accurate tables with which it abounds, will greatly enhance its value.

After we had written our review of Dr. Collins' Treatise, we received the observations recently published by Dr. Hamilton, the present distinguished Professor of Midwifery in the University of Edinburgh. The subjects treated in this volume are prolapsus, and polypous excrescence of the uterus, enlargement of the ovary, the signs and duration of human pregnancy, and the management of the three stages of labour.

It will be useful shortly to discuss the author's sentiments on some of these topics; what relates to the management of labour we shall incorporate in our analysis of Dr. Collins' work,

*Prolapsus of the Uterus.* Dr. H. finds fault with almost all obstetrical writers for their attributing prolapsus of the uterus to a relaxation of the ligaments which support it. In every case of prolapsus he says that "the vagina, or bladder, or rectum, or muscles lining the pelvis, or filling up its outlet, are debilitated or lacerated, and that the relaxation of the uterine ligaments is the effect of the prolapsus and not the cause." He condemns the practice of confining patients to the horizontal position, which is apt to injure the general health, and to increase the relaxation of the natural supports of the womb; he regards astringent injections as worse than useless; and the following very lucid objections are urged against the employment of pessaries: they act only as palliatives; they irritate the vagina and keep up a mucous discharge; they make injurious pressure on the contents of the pelvis; they are apt to become incrusted with a calcareous matter, if not frequently taken out and cleaned; and lastly, "they subject the patient to a charge of the medical attendant for life!!" We shall not mention a most ingenious method of curing prolapsus uteri once suggested but afterwards abandoned by the Professor, and which consisted in endeavouring to excite an adhesive inflammation of the vagina, so as to agglutinate its opposing surfaces; nor need we describe the particulars of a case, in which a pessary made its way into the rectum, and the result of which case finally determined him to banish pessaries for ever from his practice! The only instrument which Dr. Hamilton has used for a number of years past, is either a strong *T* bandage, or, in more serious degrees of the disease, a circular metallic belt, like that of the common truss, provided with a cross or perpendicular strap, and to this strap is attached a cushion stuffed with horse-hair, about six inches in length by three in breadth, and of a thickness proportionate to the degree of relaxation, and therefore of the support required.

"This bandage is to be worn whenever the patient is out of bed, as long as any symptom of the disease is perceived. It effectually relieves the unpleasant feelings, while it enables the patient to take walking exercise, which is so essentially necessary to the relief or cure of the disease." 29.

By this simple means, in conjunction with the use of cold bathing, and of appropriate constitutional treatment, Dr. H. has for many years past quite superseded the employment of pessaries.

*Enlargements of the Ovary.* Dr. Hamilton recognizes six varieties of ovarian enlargement; viz. a fibrous texture without any cavity, constituting a parabysma—an accumulation of fluid within the proper coat of the ovary—an accumulation of fluid, of different degrees of consistence, in cysts or sacs—a collection of hydatids, of various sizes—a complication of fatty tumors with hydatids; and, lastly, indurations like scirrhosities (we may add, medullary or cerebriform sarcoma), with cysts or hydatids.

The diagnosis of ovarian enlargement is, it is well known, seldom possible in the early stage of the disease, and, even in the more advanced, it is often extremely difficult. Not to mention pregnancy and ascites* among the states which have been confounded with it, Dr. H. informs us that he has met with several cases, in which tubercles of the mesentery or of the peritoneum, scirrhosity of the pylorus or of the caput coli, and even collections of indurated fæces in that portion of the intestinal canal, have simulated all the appearances of ovarian disease, and have misled the most experienced physicians. In reference to a case, where it was supposed that the patient was suffering from an extra-uterine enlargement, Dr. H. states:

"He was assured that the head and chest of the infant could be plainly distinguished, but that the limbs could not be felt. On examination, he found a large indurated tumour, very much of the shape which had been described, situated in the right iliac region, and reaching as low as the groin. It was exquisitely painful to the touch, and the poor woman appeared in the last stage of marasmus. He had no doubt that it was a diseased ovary, and that the sufferings of the patient admitted only of palliatives.

Death relieved the poor woman in the course of a few days, and it was then found, to the great surprise of those assembled to witness the dissection, that the disease was scirrhosity of the pylorus of great magnitude, and which had actually fallen down as low as the groin." 80.

Some forms of ovarian enlargement, even when the disease has existed long, neither induce any irregularity of the catamenial secretion, nor prevent impregnation.† Dr. H. has known more than one case, where the

---

* Dr. H. asserts that he is able to distinguish unequivocally ovarian from ascitic dropsy after paracentesis, by the following signs: "the peculiar appearance of the fluid, which in dropsy of the ovarium is commonly amber-coloured, and of the consistence of melted calf's-foot jelly, but more particularly the collapsed sac, distinctly perceivable on the day after tapping, like the contracted uterus on the day after delivery, afford certain evidence of dropsy of the ovarium." On other subjects, as well as on this, our author's language is far too peremptory, and, if unreservedly believed, might mislead an inexperienced practitioner. Dr. H. has surely the bump of self-esteem very large, and that of caution, so characteristic, it is said, of Caledonian crania, of passing small dimensions.

† Dr. Ashwell makes the same remark in reference to tumors growing from ovary was (or presumed to be) enlarged considerably during the early period of gestation, and the tumor quite disappeared after delivery.

The treatment of ovarian enlargements is necessarily, from the obscurity of their diagnosis, and the impossibility of determining their structure, unsatisfactory, and in most cases of no avail. Our author, however, assures us that, by a long-continued use of the following means, he has frequently succeeded in effecting a cure :—He recommends that moderate and equable pressure of the abdomen be made with a bandage; that the enlarged part be subjected twice a-day to percussion ;* that a course of small doses of muriate of lime (a drachm twice a-day of a mixture of seven parts of the solution of the muriate, and one of tincture of columba) be continued for at least several months, and that the patient should use daily the warm bath, and take regular exercise in the open air when the weather permits. In some cases, the Harrowgate waters, the muriated tincture of iron, the use of the conium maculatum, internally as well as externally as a cataplasm, (made of one part of the leaves, and two of linseed meal), and fomentations of the abdomen for an hour, evening and morning, with a strong decoction of the common groundsel, Senecio vulgaris (a plant formerly in high estimation, and now far too much neglected), have, in Dr. Hamilton's practice, seemed to dissipate the ovarian swelling. In conclusion we need scarcely say, that so experienced and judicious a physician as Dr. H. condemns all surgical attempts to effect a radical cure of the disease by injection, by seton, or by the still more unjustifiable operation of excision.

The leading object of Dr. Ashwell's paper, is to point out the propriety of inducing premature labour (in the sixth or seventh month), in cases where pregnancy is complicated with any large tumor in the abdomen or pelvis, whether it be connected with the uterus itself, or with the ovary, fallopian tube, or other viscus. The severe pressure and contusion, to which such tumors are exposed from the unyielding gravid uterus in the latter months of pregnancy, are apt to excite them to rapid increase of size and to changes of structure, which generally compromise the safety of the mother.

From the subject of enlargements of the ovary, Dr. H. passes to that of the *signs and duration of human pregnancy*. It is not our intention to discuss these topics at any length, having within the last year or two devoted more than one ample article to their illustration ; but as our readers may naturally be anxious to be made acquainted with the sentiments of our author, than whom perhaps none has had a wider experience in obstetrical practice, and whose observations must therefore necessarily influence the belief of a great mass of those who will peruse his book, or at least our review of it, we think it right to submit them to their notice.

It would have been pleasing to us to have been able, in the discharge of our duty as faithful critics of the medical press, to approve and sanction these

---

the uterus itself; often, " menstruation is almost as regularly performed as when the uterus is not structurally diseased."

* The percussion may be made either with the fingers, or with an instrument contrived for the purpose by Dr. H. and which consists of five metallic balls, imitating the points of the fingers, affixed to as many rods which spring from the handle.

sentiments; but this we cannot conscientiously do, despite the high authority of the Professor's name. Throughout both chapters, there is so much of unproved and dogmatical assertion—of self-confident and overweening prejudice, and of unfair, nay almost ungenerous incredulity of the statements and observations of former writers, that Dr. Hamilton himself cannot be surprised, if others withhold their full assent to his own very positive and peremptory precepts. Our readers may judge for themselves, when they have read the following announcements:—

Dr. H. repeatedly asserts, that there are "invariable signs" and "unequivocal marks" of the existence of pregnancy, in its early as well as in its later stages. "There are (says he) two circumstances which invariably attend pregnancy during the early months, viz. suppression of the catamenia, and a perceptible change on the surface of the mammæ surrounding the nipples." He admits that, in some pregnant women, there may be periodical bloody discharges from the vagina; but he qualifies this admission by stating, that there is always an irregularity in the date of the recurrence (though, perhaps, to the extent only of a day or two), as well as in the duration, the quantity, and the quality of these discharges. He may well add, "in all doubtful cases, accurate information on these points ought to be obtained;"—the very difficulty consists in obtaining such accurate information. Let it not, however, be supposed that we differ in opinion with the Professor on the point at issue; our well-assured conviction is, that the *genuine* menstrual flow does not, and cannot, take place during pregnancy; but of this we are equally certain, that not one woman in a thousand can distinguish *it* from other sanguineous discharges; and it is on their testimony that the accoucheur must in general found his opinion. According to Dr. Hamilton, the other invariable sign of early pregnancy is the change in the appearance of the areolæ round the nipples. To avoid all misconception, we shall quote his own words:

"For many years, the mark on which he has placed his principal reliance for distinguishing the true areola consequent upon impregnation, from the appearance of the surface of the mamma peculiar to the individual in the unimpregnated state, is a certain degree of turgescence on the surface of the discoloured ring, which becomes more and more distinct towards the latter end of pregnancy. In women who have had a family, and in whom there is a brown or dark areola while not pregnant, there is no sensible turgescence of the surface; but during pregnancy the turgescence alluded to is, in most cases, evident to the naked eye, and, according to the author's experience, can in all cases be detected by a magnifying-glass. The elevation of its outer surface can be thus seen distinctly. During menstruation, indeed, there is a certain degree of turgescence, but it proves of temporary duration only, and when the conception becomes blighted in the early months, the turgescence gradually disappears." 146.

Our own experience, and, we may add, that of almost all the latest and best writers on midwifery and on medical jurisprudence do not certainly warrant that unqualified and unequivocal reliance on the changes of the areolæ, as a sign of early pregnancy, which Dr. H. puts in them. Equally unsatisfactory to us has been, in many cases, the test, which, it would seem, has never failed or misled Dr. H., of pregnancy during the latter four months. The progressive increase of enlargement of the belly, and the secretion of milk in the mammæ, he admits to be fallacious signs:

"But the movements of the infant being independent of the control of the patient, can always be distinguished by an attentive practitioner, and in proportion as pregnancy advances, the facility of this detection increases."

And he adds :

" For the first month after quickening, if the hand be applied over the region of the uterus, the movement of the infant feels like that of a ball suddenly rebounding from the part on which it is thrown, but after another month, when the infant moves, a bulky body can be perceived as if starting, and during the last two months, besides the starting, the infant can be felt to move occasionally its several limbs." 148.

Dr. H. like most physicians of the old school, has no faith in, and indeed no experience of, Auscultation, as affording any indication of pregnancy. He has never tried it, " for the plain reason, that he has not met with a case during the last thirty years where he could not ascertain pregnancy after the fifth month (where the infant continued to live), by the marks suggested in the preceding observations." We shall however allude to some remarks which he offers on this subject in a future part of this article, and we will therefore now pass to another topic on which he is more at home—we mean the duration of human pregnancy.

Contrary to the opinion of the leading accoucheurs in London, most of whom, in their evidence some years ago on the case of the Gardner Peerage, agreed that pregnancy does not exceed the period of nine calendar months or forty weeks (counting from the day preceding that on which the menses ought to have appeared, had the woman not conceived), Dr. Hamilton " is quite certain that the term allowed by the Code Napoleon, viz. three hundred days, is too limited, and he is inclined to regard ten calendar months, which he believes to be the established usage of the Consistorial Court of Scotland, as a good general rule."

We now proceed to the more immediate subject of this article—the consideration of the duties of the accoucheur in the various kinds of labour,—the natural, the preternatural or difficult, and the complicated,—founded on an analysis of Dr. Collins' treatise.

*Natural Labours.* Dr. C. recommends as a general rule, that every patient should, on the occurrence of the very first symptoms of labour, take an aperient, so as to effectually empty the bowels. The advice is, on the whole, a good one. The passage of the child is facilitated by the empty state of the rectum, and the feverishness of the system also is kept under. Whenever the labour is protracted, the accoucheur ought to enquire repeatedly as to the state of the bladder. A large and stimulating enema, thrown up every five or six hours, will often supersede the necessity of using the catheter. The puncturing of the membranes in natural labours, is very rarely required ; it is to be done only when all the parts are well dilated, and the delay arises from a want of proper energy in the uterine action. We need not dwell on the directions (they are all simple and judicious) for the management of natural labours, which Dr. C. inculcates on his readers.

The most important is that, wherein he so earnestly explains the necessity of promoting the complete contraction of the uterus after the expulsion of the child. Throughout the work, he repeatedly urges the precept, that we should " allow the uterus slowly to empty itself, leaving the body and limbs of the child to be expelled by uterine action only; and by pursuing the fundus uteri, in its contraction, with the left hand placed on the abdomen, not only until the fœtus be entirely expelled, but continuing this pressure steadily afterwards, so as to keep the uterus in a state of permanent contraction." The paramount value of this rule must be appreciated by every experienced accoucheur. Let the young practitioner, therefore, never omit to follow it, as it affords the only sure safeguard against that frightful accident—flooding. If it was less frequently neglected, we should hear of fewer accidents after delivery.

"As soon as the head and shoulders are expelled, the left hand is to be instantly placed on the abdomen, immediately above the fundus uteri; thus following down, by a steady pressure, this organ as it contracts, so as to bring it as low into the pelvis as practicable, without using violence. We keep up this pressure *most diligently* from five to ten minutes after the feet are expelled, and when the womb feels firm and well contracted, we make the nurse-tender supply our place, until the child is separated and the binder put on." 122.

In all labours, whenever the head is expelled, the accoucheur ought immediately to ascertain whether the cord is around the neck of the child or not.

The chapter on the subject of *tedious and difficult labours* is full of admirable advice. Dr. C. briefly alludes to the most frequent causes—imperfect uterine action, rigidity of the os uteri and other soft parts, adhesions of the vagina,* early rupture of the membranes, disproportion between the size of the child's head, and the mother's pelvis, malformations,—congenital or from disease,—of the child, &c.

Dr. Hamilton has made some valuably practical remarks on the dangers to be apprehended from allowing the first stage of labour—" all that happens previous to the complete dilatation of the os uteri"—to be protracted too long. His great experience and eminent success entitle his remarks to be well considered by every accoucheur. We shall, therefore, condense all the useful matter contained in the chapter, " on the management of the first stage of labour." The rule which he inculcates on his pupils, and which, he tells us, he has invariably followed for the last 35 years in his practice, is that, " whenever there are regular pains—to be distinguished from what are called false or spurious pains, by their recurring with regularity, and by being accompanied with a tightening of the edges of the os uteri—the first stage of labour should be completed within *twelve* or *fourteen* hours from its *real* commencement."† When protracted beyond this period, the accoucheur has reason to apprehend the following dangerous consequences: the powers of the uterus may be so exhausted, as to be inadequate to expel

---

* Dr. C. alludes to four cases of this nature, in which adhesion, the consequence of previous difficult labour, had taken place to such an extent, as to form an unyielding band in the vagina, which completely resisted the descent of the head. Dr. C. divided the band laterally with a probe-pointed bistoury, and in all the cases the labour terminated quite favourably.

† It is well known that sometimes, after regular pains have commenced, there is a complete suspension of them for some hours. " If, during this interval (says Dr. H.), there be no injurious pressure upon any part of the mother, the previous pains are not to be reckoned; but the duration of the first stage is to be dated from the recurrence of the pains."

the child with safety; the infant may be destroyed by the continued pressure of the uterus on its navel-string; the uterus is apt, after the expulsion of the child, to contract either irregularly or incompletely, and thus occasion a retention of the placenta or a dangerous hæmorrhage; and, lastly, the mother is rendered more subject to dangerous febrile and inflammatory affections.

Dr. H. illustrates his precepts by adducing several cases; and he alludes in a pointed manner to the melancholy death of the late Princess Charlotte, which, he thinks, was attributable to Sir Richard Croft having allowed the first stage of labour to be unnecessarily prolonged. It appears that the pains commenced at seven o'clock, p.m. of Monday, Nov. 3d, 1817, and that it was not till three in the afternoon of Wednesday, that the head of the child cleared the os uteri. At six o'clock of that afternoon, the meconium of the infant was discharged—a clear evidence that its life was in jeopardy—and, at nine o'clock, a still-born infant was expelled. The placenta was retained by the hour-glass contraction, internal uterine hæmorrhage followed, and the Princess sunk in little more than two hours after delivery.

By adopting the rule which we have explained, Dr. H. assures us that no patient under his charge for the last thirty-five years has been above 24 hours in labour, and, excepting in cases of disproportion, none so long. He animadverts, with censure, on the published statements of several of his distinguished cotemporaries, as Dr. Ramsbotham, Dr. D. Davis, Drs. Maunsell, Beattie, and Collins, of Dublin, and Madame la Chapelle, of Paris, who seem to allow the first stage of labour to be often prolonged greatly beyond 12 or 14 hours, and delivery to be protracted sometimes to 36, 48 hours, and upwards. Dr. Burns, of Glasgow, certainly one of the ablest writers of this or any age on midwifery, has for many years past been a convert to Dr. Hamilton's opinions. In the earlier editions of his work he had stated :—" It is impossible to determine how many hours a labour may be permitted to continue; for time alone is not to be our rule—we must be regulated greatly by the effects of the labour;" but, in all the later editions, he has modified very materially this statement, as will appear by the following extract from p. 411 of the eighth edition.

" The first stage of labour ought always to be accomplished within a certain time, varying somewhat according to the constitution of the patient and the degree of pain. If the pain be continuing without suspension, or an interval of some hours, and the labour be going on all the time, but slowly, it is a good general rule to effect the dilatation of the os uteri within ten or twelve hours at the farthest from the commencement of regular labour." 208.

The testimony of so excellent an accoucheur as Dr. Burns will have its due weight with the young practitioner.

It will now be asked—what is the treatment recommended by Drs. Hamilton and Burns, to prevent the undue prolongation of the first stage of labour? and we proceed, therefore, to offer a few remarks on this subject.

In a large proportion of cases, venæsection will be found to furnish the most effectual means of promoting the dilatation of the os uteri. This remedy is applicable more especially to all cases where the parts are rigid and unyielding, as often happens when the membranes have prematurely burst, and also in first labours. The use of an opiate enema after the bleeding will be found to be in many cases an excellent adjuvant. When it is not deemed expedient to have recourse to venæsection, the opiate enema alone will generally succeed. In cases of tedious dilatation of the os uteri, in which bleeding and anodynes are not advisable, gentle manual assistance may be given not only with perfect safety, but with the happiest effects. As it is of the highest importance that the accoucheur should rightly understand the kind of assistance to be given, and the circumstances under which it is proper, we cannot do better than submit the sentiments of two such eminent men as Dr. Burns and Dr. Hamilton to the consideration of our readers.

"If," says Dr. B. "the os uteri be lax, and thin or soft, it is both safe and advantageous to dilate it gently with the finger during a pain. If this be done cautiously, it gives no additional uneasiness, whilst the stimulus seems to direct the action of the uterine fibres more efficiently towards the os uteri, which sometimes thus clears the head of the child very quickly, and the pains which formerly were severe, but in the language of the patient unnatural, and doing no good, become effective and less severe though more useful." 205.

He deprecates all forcible and irritating dilatation, so as to occasion any pain, and cautions his readers against continuing it too long, or repeating it too frequently. Dr. Hamilton remarks:—

"It is done best by pressing on the anterior edge of the os uteri during a pain, with two fingers, with such moderate force as shall not give additional pain, and shall appear to excite the natural dilatation as much as to produce mechanical opening. By doing this for several pains in succession, or occasionally during a pain, at intervals, according to the effect produced, and the disposition to yield, we shall soon have the os uteri completely dilated. 206.

If the os uteri be somewhat projecting, the accoucheur may now and then introduce two of his fingers within it, and extend them laterally with gentleness during a pain, taking care all the while not to press against the bag of the waters.

In some women there is such a relaxation of the vagina, and of all the parts lining the pelvis, that the uterus is forced during every pain low down upon the external parts; and this state of things may occasion great delay in the dilatation of its orifice. Under these circumstances it is only necessary to support and retain the uterus in situ during the pains, by applying two fingers to the edges of the os uteri.

There is one cause of undue protraction of the first stage of labour—fortunately of not frequent occurrence—which Dr. Hamilton ascribes to the existence of an undeveloped band of fibres surrounding the cervix uteri.

"This cause of protracted labour," he says, "can be detected without difficulty, for the edges of the os uteri swell during the pain, as if distended with air, becoming relaxed when the pain ceases; and notwithstanding strong labour throes, neither the membranes nor the infant are brought in contact with them. If, during the interval of the pains, the finger be carried up within the os uteri, the stricture of the cervix will be distinctly perceived." 217.

The treatment which Dr. H. recommends in such a case consists in drawing blood, if the state of the patient will permit, in exhibiting an opiate enema, and after this in introducing the finger above the stricture, and in pressing from within outwards on the resisting band of the uterus during each successive pain.

The last clause of the Professor's advice must be acted upon with great circumspection and gentleness. We are great enemies to all unnecessary manual interference in the management of labour, and we should be rather unwilling to carry the finger within the cervix uteri, above the seat of the stricture, for the purpose of pressing upon it from within. We prefer the method recommended by Dr. Burns, of making the pressure merely on the anterior edge of the os uteri, as already explained in an extract given above.

So much for the management of the first stage of labour, when there is any unusual delay in the dilatation of the os uteri. The chapter on this subject contained in Dr. Hamilton's book is certainly useful, as our readers may judge by the topics which we have selected from it. We wish that we could with propriety say as much for the succeeding chapter, "On the Management of the Second Stage." Thirty pages of print to be occupied with recommending the accoucheur to apply his *naked* (without a napkin) hand to the perineum when the head is pressing down, and to smear the parts well with soft lard! In truth this is the sum and substance of the whole chapter!!

The chapter on the management of the third stage—by which is meant the separation and expulsion of the placenta and membranes—is rather better, but still far too prolix. We have incorporated all its valuable contents in our remarks on the retention of the placenta.

We now return, after this lengthened interpolation, to our running analytic commentary of Dr. Collins's observations on the treatment of painful and difficult labours. We are to suppose that the head of the child, having cleared the orifice of the uterus, and descended into the pelvic cavity, is there detained so long, that the accoucheur deems it expedient no longer to trust to the natural efforts, but to resort to manual interference.

With respect to the proper period of the labour, at which the accoucheur may be required to give instrumental assistance, he should be regulated much more by the existing state of his patient, than by the mere duration of the pains. Some women will suffer more in thirty hours than others in 90.

"Generally speaking, so long as the pulse remains good, the bowels and bladder act well, the soft parts remain free from severe pressure, and uterine action continues, so as to cause the presenting part to descend ever so slowly, the patient having no pain in the abdomen on pressure, or local distress, the child at the same time being alive, as indicated by the stethoscope, I am satisfied, no attempt should be made to deliver with instruments, and that he who does so, wantonly exposes both mother and child to danger." 8.

The assistance of a second physician should be in every instance, where practicable, obtained, before the forceps is applied. The application of the instrument indeed is almost always abundantly easy, under the only circumstances in which such assistance is required,—"when the os uteri is fully dilated, the soft parts duly relaxed, the head resting on the perineum, or nearly so, and the pelvis of sufficient size to permit the attendant to reach the ear with the finger;" and be it remembered that, when all these favorable conditions are present, unassisted uterine action seldom fails of expelling the child. How rare must be the necessity for instrumental assistance, when we learn that in 16,414 deliveries in the Dublin Hospital, it was rerequired 27 times only—the forceps 24 times, and the lever thrice—and that in 13 of these 27 cases, the labours were *complex*. The entire number gives us the average of about 1 in 608 deliveries. "According to this calculation, most physicians in private practice should require to use the forceps or lever but very seldom; as supposing an individual to attend 4000 cases in the course of his life—which is a greater number than falls to the lot of most men—instruments would be necessary in little more than *six* cases." Dr. C. unhesitatingly condemns the use of the forceps, when the head becomes fixed or impacted in the pelvis, and when the ear cannot be reached with the finger except by violence, in consequence of any disproportion between the head and pelvis, whether this arise from the increased size of the former, or from the diminished dimensions of the latter.

It is better, if instruments must be used under these circumstances, to sacrifice the life of the child by resorting to embryotomy, than to endanger that of the mother by rash and violent efforts to extract it with the forceps.

"The results I have witnessed (we quote from our author), from such practice, were most distressing; in some, the neck of the bladder, or urethra, either lacerated, or the injury by pressure from the forceps so great, as to produce sloughing, and consequent incontinence of urine: in others, the recto-vaginal septum destroyed, either of which, renders the sufferer miserable for life; and in two cases, where the mouth of the womb was imperfectly dilated, so much injury inflicted on this part, as to terminate in death." 13.

Drs. Denman, Burns, and Merriman, are equally strenuous in condemning the use of the forceps, unless the ear of the child can be distinctly felt, and the perineum be sufficiently relaxed. To talk of using, as a general practice, the forceps in preference to the crotchet, implies a most pernicious and dangerous doctrine; for in truth the one instrument can seldom or never be safely substituted for the other. When the case is one in which, according to the rules now explained, the forceps is applicable, no reasonable man would ever think of using the perforator; and "he who uses the forceps *under other circumstances*, will find reason to regret much he had the misfortune to do so, and his patients will regret still more they had the misfortune to employ him."

Whenever the disproportion between the head and the pelvis is so great as to prevent the physician reaching the ear with the finger, and the life of the mother is endangered by any further delay, the only safe means of effecting delivery is the operation of embryotomy. The following directions are full of practical interest.

"When uterine action continues regular and strong, for 12 or 24 hours after the os uteri is dilated or nearly so, without the child's head making progress; it being firmly compressed in the pelvis, not leaving space for the introduction of the finger to feel the ear, or in some the passage of a catheter into the bladder; the urine perhaps retained from severe pressure on the urethra; or when removed, bloody or very scanty, and of a deep colour; the patient complaining of acute pain on pressure, in any part of the abdomen, the pulse being at the same time hurried, and the strength failing; these are symptoms indicating the use of the perforator, and their being *urgent* or *otherwise* should make us deliver sooner or later." 17.

Dr. C. is of opinion that the circumstance of the head remaining *quite stationary* after the dilatation of the os uteri for a certain number of hours, although the pains have continued all the time, is a surer proof of some impassible obstruction than any which can be derived from the most accurate manual examination.

As long as the head advances *ever so slowly*, the patient's pulse continuing at the same time good, the abdomen free from pain on pressure, and no obstruction existing to the withdrawal of the urine, embryotomy ought not to be thought of, unless the child be dead. Every one knows what a difficult and important problem in midwifery it has ever been, to determine previous to undertaking this distressing operation, whether the fœtus is alive or not. Before the introduction of auscultation, the signs chiefly trusted to were often uncertain and misleading. Dr. C. gives his unqualified testimony to the invaluable services which Laennec's discovery has bestowed upon the obstetrical art.* How often would the physician be justified in delivering a woman by embryotomy, and thus be enabled to save her hours and even days of the most painful suffering, if he was assured of the death of the fœtus!

There is much to reflect upon in the following short sentence:—

"I have often known patients permitted to endure the most urgent distress for a period of 40, 50, 60, or 70 hours; and at the expiration of this time of sorrow, giving birth to a child, evidently dead for hours, and occasionally putrid." 19.

Four cases in illustration are recorded. Our readers must consult the work itself for the particulars. In one case the poor woman had been *three entire days* in dreadful suffering, and at length was delivered of a *putrid* child. Need more be said of the inestimable advantages of any diagnostic means, which could have saved even one fellow-being from such protracted agony? Although auscultation affords by far the most satisfactory guide to determine whether the fœtus be alive or not, it is pleasing to be furnished with other means and other rules to assist our diagnosis on this point, before having recourse to the perforation of the head.

A volume of practical wisdom is contained in the following extract:—

"I have no difficulty in stating, and that after the most anxious and minute attention to this point, that where the patient has been properly treated from

* We have already noticed that Dr. Hamilton is no disciple of auscultation. Most of his objections are indeed very trifling; but there is one which deserves notice from its startling novelty. Dr. H. says that in his long experience he has in no instance found the pulse of the child, before it begins to breathe, to exceed sixty beats in the minute. Of the truth of this, he has repeatedly convinced himself by counting the pulsations of the cord not only while the child was still in utero, but also after its expulsion, before respiration commenced. The experience however of his friend and coadjutor Dr. Moir (and we need scarcely add, of all other accoucheurs) is directly opposed to the Professor's statements.

In several cases, where turning was necessary, Dr. M. found that the pulsations of the fœtus in utero were 120 and upwards, and moreover that the number of pulsations ascertained with the hand corresponded exactly with those which he discovered by means of the stethoscope applied to the abdomen of the mother. It deserves however to be mentioned that Dr. Moir invariably found that the number of the fœtal pulsations diminished greatly (sometimes from 120 to between 60 and 70) whenever uterine contraction supervened, and again increased, whenever the contraction was over. This circumstance is the more worthy of being noticed, as Dr. E. Kennedy has not alluded to it in his excellent work, and the reverse of it is maintained by Dr. Hohl, whose work we reviewed in the Foreign Periscope of our last number but one.

The assertion of Dr. Hamilton that the pulse of the child immediately after delivery, provided respiration has not begun, is generally sixty or thereabouts is surely quite incorrect.

the commencement of her labour; where strict attention has been paid to keep her cool; her mind easy; where stimulants of all kinds have been prohibited, and the necessary attention paid to the state of her bowels and bladder; that, under such management, the *death* of the *child* takes place in laborious and difficult labour, before the symptoms become so alarming, as to cause any experienced physician to lessen the head. This is a fact, which I have ascertained beyond all doubt by the stethoscope; the use of which has exhibited to me the great errors I committed, before I was acquainted with its application to midwifery, viz. in delaying delivery, *often,* I have no doubt, so as to render the result *precarious* in the *extreme,* and in some cases, even *fatal.*" 16.

As affording a fair estimate of the frequency of impracticable labours requiring the use of the perforator, the tables of Dr. C. shew that in 79 of 16,654 births delivery was effected by lessening the head, in consequence of tedious and difficult labour; exclusive of 39 other cases where either the uterus was ruptured, or the umbilical cord was prolapsed and pulseless, or where the patient was labouring under convulsions;—the total number being 118, giving an average of 1 in 141. In six of the 79 cases the forceps had been ineffectually tried beforehand. Fifteen of the women died: in several of the fatal cases, the patients had been in labour for a great length of time, and when admitted into the hospital, were in a state of complete exhaustion. The dissection in one of these distressing cases shewed that the neck of the bladder and also the vagina were completely eroded and in a state of gangrene—the results no doubt of the prolonged pressure of the impacted head. Seven of the fifteen children were born *putrid.*

On the subject of *Presentations of the Face and with the Face to the Pubes,* we are furnished with the following data. In 33, of the 16,654 births the face presented: in all, delivery was effected *without assistance.* In 12, the head presented with the face turned to the pubes: in four of these the use of the crotchet was necessary;* not however in consequence of the mere irregular position of the head, but of co-existing malformations of the pelvis, or of the cord and hand having descended along with the head, and the pulsation in the former having ceased. The following directions will be read with interest by the practical accoucheur.

---

* It appears that the crotchet is employed more frequently, and the forceps or vectis much less frequently in the Dublin Lying-in Hospital, than in most similar institutions. The following Table will shew the practice of different accoucheurs in reference to this important subject.

| Physicians. | Total number of births. | Forceps or vectis used in. | Crotchet used in. | Relative frequency of instrumental deliveries |
|---|---|---|---|---|
| Dr. Carus of Dresden.... | 2549 | 184 | 9 | 1 in 13 |
| Dr. Kluge of Berlin, .... | 1111 | 68 | 6 | 1 in 15 |
| Dr. Nægele of Heidelberg | 1711 | 55 | 5 | 1 in 28 |
| Dr. Boer of Vienna...... | 9589 | 35 | 13 | 1 in 199 |
| Mad. Boivin of Paris* ... | 20517 | 96 | 16 | 1 in 183 |
| Dr. Merriman.......... | 2947 | 21 | 9 | 1 in 98 |
| Dr. Joseph Clarke ...... | 10199 | 14 | 49 | 1 in 162 |
| Dr. Collins ............ | 16654 | 27 | 118 | 1 in 114 |

* The mortality at the La Maternite in Paris is "vastly beyond what we are acquainted with elsewhere."

"Where the face is turned to the pubes, the perinæum is greatly distended during the passage of the head, in some instances, so as to endanger its safety; in such, the distention may be much relieved by passing up the finger and freeing the chin, so as to cause it to escape the arch of the pubes. All attempts to alter the position of the head in the early period of labour, when found presenting in either of the above-mentioned ways, are, in my opinion, injudicious. The labour should be treated as one perfectly natural." 35.

And he adds "the position (whether the face presented, or was turned to the pubes) of the child did not in any instance render artificial delivery *necessary*."

On the treatment of *Breech Presentations*, the remarks of Dr. Collins are pervaded with the soundest advice. He strongly urges the importance of conducting the early stage of the delivery, before and during the expulsion of the breech, slowly and very gradually, so that the parts may be well relaxed and expanded when the head comes down, Whenever we have reason to suspect a breech or foot presentation, we should be more than ordinarily cautious not to rupture the membranes. No assistance is usually required, except in supporting the perineum, until the breech and lower extremities are fairly protruded. If indeed the uterine action be from any cause inefficient, or if any accident occur rendering speedy delivery necessary, the accoucheur may by hooking his finger round one of the groins of the child assist in drawing down and expelling the breech. The use of the blunt hook for this purpose is rarely or never necessary. Dr. C. has employed it only *once* in upwards of 24,000 deliveries.

When the breech and lower extremities are expelled, the finger should be passed up the vagina to ascertain the state and position of the umbilical cord, as the chief danger of these presentation-labours arises from the compression of the cord and the interruption of its circulation during the delivery of the head. If the pulsations of the cord be feeble, or if we have other reasons to suspect that the life of the fœtus is endangered, the delivery ought to be expedited as much as the mother's safety will permit, by cautiously bringing down the arms, the one after the other, and then by passing the left fore-finger into the child's mouth, so as gently to depress the chin on the sternum, we are able to direct the efforts of our right hand at extraction. Should any considerable obstruction to the expulsion of the head be experienced, no motive must ever induce the accoucheur to use *violence*, for the mother may be irretrievably injured, and, moreover, the chances are that the life of the child will not be saved. Whenever the head is strongly and for any length of time impacted, we may be almost assured, says Dr. Collins, that the child has ceased to live, in consequence of the circulation through the cord having become interrupted.

"The only safe plan, under these circumstances, will be to lessen the head by means of an opening made behind one or both ears. This expedient, however, is *very seldom* necessary, provided the early part of the labour be not hurried, or the pelvis not much under size. We only met with *one* case requiring this method of delivery." 43.

The actual number of breech-presentations in the practice of Dr. Collins, was 242 out of the 16,654 births (not including those which occurred in twin cases). Of these 242 children, 73 were still-born, of which 42 were putrid. Of feet-presentation cases there were 127; in 62 of which the children were dead; and of these 62, 41 were putrid.* Before quitting the subject of breech and foot presentations, it may be useful to remind the reader of the propriety of his acquainting the friends of the patient with the risk to which the child is exposed, as soon as he has ascertained the presenting part. As a matter of course, it should not be communicated to the woman herself.

*Presentations of the shoulder* or arm are much more serious accidents than those which we have hitherto examined, the life of both mother and child being but too often endangered.

The importance of ascertaining exactly the nature of the presentation at an early period of labour, and before the bag of the membranes has burst, cannot be too earnestly urged on the attention of every accoucheur. We may assume it, as a position of general truth, that when the shoulder or arm presents, the delivery of the child cannot be effected without the interference of art: either the child must be turned and the case be converted into a footling one, or when this is impracticable, the perforator must be resorted to. Dr. Denman's doctrine of "spontaneous evolution" is too fanciful a hypothesis to base any practical directions on it. Out of nearly 35,000 deliveries in the Dublin Lying-in Hospital, *one* case only (and that of very questionable authenticity) of spontaneous evolution is on record.

Although we are not prepared to assert that a shoulder or arm presentation is never rectified by the efforts of unassisted Nature, we think it better to exclude the consideration altogether from our minds, when we have to determine on the line of practice to be followed in the emergency to which our present remarks apply.

"The propriety" says Dr. C. "of turning the child, when it presents with the shoulder, or arm, in all cases *where it can be effected with tolerable safety to the mother*, cannot be questioned."

*This* is to be the guiding rule of the accoucheur. It is alike simple, unerring, and imperative. One of the most important objects to be determined, is the proper period at which the operation of turning may be attempted. If the os uteri be fully expanded, if the parts be relaxed and soft, and the parturient pains be not violent, we may safely proceed to turn the child.

The attempt is greatly facilitated, when the membranes are entire, or have but lately burst, and when a portion of the liquor amnii is still present in the womb. Unfortunately indeed it not unfrequently happens that the membranes give way at an early stage of the labour, and that the womb in consequence has contracted firmly and almost spasmodically round the protruded part, so that although the os uteri be expanded, the finger cannot be passed between its margin and the arm or shoulder. This is a case of *difficulty*, it is true, but it ought not to be one of *embarrassment* to the accoucheur. The course he must pursue is simple and undeviating. All violence is to be most

---

* The great mortality of children born by breech or foot presentation deserves to be noticed. It is to be attributed in a great measure to the circumstance of many of the children being dead before the commencement of labour. This is proved by the number of putrid fœtuses, 83 out of 369, alluded to above. A large proportion of these births occurs prematurely, between the seventh and ninth months. Nature thus seems to have provided in some degree against the danger of preternatural presentation deliveries.

carefully avoided, and on no consideration is the hand ever to be thrust forcibly into the uterus. Delay may be attended with danger, but rashness and violence with the almost inevitable destruction of the poor patient. A bleeding from the arm, large if the patient's strength will bear it, ought to be first practised, and then repeated doses of tartar emetic may be given at short intervals, until a certain degree of nausea, and of general relaxation be induced. Dr. C. is in the habit of ordering a table-spoonful of a mixture containing four grains of the salt in six ounces of water to which 30 drops of the acetum opii are added, every half hour. These means, judiciously employed, will almost invariably produce the desired effect. Dr. C. says

"I never knew of more than one instance, where the patient could not be delivered previous to death, owing to the rigid and unyielding state of the os uteri; this occurred when I was a pupil in the Hospital; the mouth of the womb was absolutely as firm as a piece of thick leather, and embraced the arm of the child as tightly as a ligature could be applied, without cuttingthe part." 67.

To attempt turning, while the pains are still forcible and frequent, and the os and cervix uteri closely embrace the projecting part of the child, cannot be too earnestly deprecated.

The bladder is invariably to be emptied before we commence the operation. In some cases, the rectum also may require to be evacuated. The hand is then to be passed slowly, tenderly and yet firmly; never hurriedly or with any sudden thrust. Whenever a uterine pain comes on, we must wait until it subsides, laying our hand flat on the body of the child during the continuance of the pain. If the uterine action be violent, the tartar emetic mixture with a larger quantity of the acetum opii will generally be found to allay it considerably. The accoucheur must be on his guard not to mistake a hand for a foot; a mistake which he may be apt to commit, from his own hand becoming quite benumbed by the constriction of the womb.

Again we have to beseech the physician to proceed with uniform caution, gentleness and steadiness in every step of the operation of turning. Half an hour, or even a full hour may be required to complete it with safety in cases of difficulty. So much for the one method of effecting delivery in shoulder or arm presentations: the other consists in diminishing the size of the child by perforating the thorax, and then bringing down the breech. This is always a lamentable expedient, and is never to be used except when the safety of the mother demands it. Certain however we are that accoucheurs have been on the whole far too timid and reluctant in the adoption of it, and that while many a mother's life has been sacrificed to unnecessary delay, scarcely one child has been saved thereby.* Dr. Collins' sentiments deserve to be expressed in his own words.

---

* The history of the sixth case related by Dr. Ashwell affords a melancholy proof of the fatal dangers of injudicious interference. At 10 P. M. the right extremity of the child was found protruded beyond the os externum. Two rather *forcible* attempts at turning the child seem to have been made, but in vain: not even the knees or feet could be reached. After a bleeding was practised, and another large dose of opium given, a third attempt to turn was made; but this was as fruitless as the two preceding. Early on the following morning Dr. Ashwell himself visited the poor woman: "the abdomen was tender to the touch, and the vagina and internal parts were hot, tumid and sore." In this deplorable

"I shall now endeavour to point out those cases, where no prudent practitioner would, we think, attempt to turn; yet, such attempts we have witnessed, but with very fatal results. The cases we more particularly allude to, are, where the waters have been long discharged, the uterine action powerful, and the child's body forced low, and firmly impacted into the pelvis for several hours; in such, turning would be hazardous in the extreme; besides, under these circumstances, the child's life is destroyed by pressure; a fact which we have clearly ascertained by the stethoscope; by which we are enabled to detect the death of the fœtus, at a period, when otherwise, we might be induced to expose our patient to the utmost danger in the attempt to turn, where we have a comparatively safe means of delivery. The singularly rare occurrence of a living child being born, where spontaneous evolution takes place, even in the most expeditious manner, affords additional testimony of the fatality to the child in such cases." 70.

Whenever we have good reason to believe that the child is dead, we need have no scruple in resorting at once to embryotomy, if there be the slightest difficulty in turning the child. The late admirable accoucheur Dr. Joseph Clarke alludes to "many patients losing their lives by practitioners of good repute insisting on turning the fœtus, although evidently putrid;" and recommends that in such cases the thorax or abdomen should be rather perforated, and the breech then brought down by the aid of the crotchet or blunt hook.

Dr. Collins adds his powerful testimony to the same effect: "This," says he, "from ample experience is the practice we would unhesitatingly recommend; and that *in all cases* where the death of the child can be satisfactorily ascertained." The operation is sometimes very tedious and troublesome, requiring in most instances an hour and a half or two hours for its completion. Occasionally when the resistance is great, and a very speedy delivery is not necessary, it may be advisable, after perforating the thorax, and thus lessening the bulk of the child, to leave the patient quiet for a few hours: the fœtus becomes collapsed, and Nature will often complete the expulsion.

Of the 16,654 births, there were 40 in which the shoulder or arm presented. In 33 of these the children were delivered by turning: in three, however, the head required to be perforated before the delivery could be accomplished; 20, one half of the entire number, were born alive. In six of the 40 cases it was necessary to apply the perforator to the thorax and abdomen, and then to deliver by bringing down the breech. Four of the women died: two from rupture of the uterus; one from puerperal fever; and one from phrenitis.

"Of the *twenty* still-born children, *six* had the thorax lessened; *three* had the head perforated after having been turned, the pelvis being under size; *three* were putrid; *two* had the arm down on admission; with *one* the waters were discharged on admission; with *one* the waters had been 40 hours discharged; and with *one* 7 hours; the child being also very large; with *one* there was much

---

condition she was removed by his advice from her own dwelling into the hospital! At half-past one P. M. (fourteen hours and a half after the first attempt to turn) Dr. A. endeavoured to decapitate the child, but he was foiled. At five P.M. and again at ten P.M. he made two other attempts, but was equally unsuccessful, the conjugate diameter of the brim of the pelvis being scarcely more than two inches and a half. The woman died undelivered a few hours afterwards.

difficulty in getting away the head, the bones of which were much compressed; with *one* the placenta presented; and with *one* there was neither delay nor difficulty.

This short abstract is given, to shew, that, in the great majority of these cases, death had taken place previous to any attempt to deliver; and that in *none*, was the actual deliuery the causr of deatb, where disproportion did not exist." 74.

The subject of "*uterine hæmorrhage,*" *or flooding*, next demands our attention. It is certainly one of the most alarming and dangerous accidents to which parturient women are exposed. Alarming, indeed, it is to the physician also; but let us remember that on no occasion, within the whole range of medical practice, may the utility of our art be more indisputably vindicated, than by the successful treatment of hæmorrhage during or after labour. The classification of uterine hæmorrhages into those which are *unavoidable*, and those which are *accidental* (according as the placenta is attached over the os uteri, or at some other part of the womb), first distinctly urged and illustrated by Mr. Rigby of Norwich, must ever be borne in mind, as the success of our practice will in most cases mainly depend on the correctness of our diagnosis. If we have once fairly ascertained that the placenta is situated directly over, or with a projecting edge at the os uteri, we are quite certain that hæmorrhage, if it has not already occurred, must take place when the efforts of dilatation commence. It is therefore aptly denominated "unavoidable."

With respect to the assistance which is demanded from the medical attendant under such circumstances, fortunately there cannot be any difference of opinion, except perhaps in reference to the exact time, at which his interference is most proper. The amount of the hæmorrhage is by no means always proportionate to the extent of the separation of the placenta from the uterus: unhappily too it may be very large, even when but little escapes outwardly, for the blood may be poured into and retained within the uterine cavity. It is also to be remembered that some women cannot bear any profuse loss of blood, without an almost immediate sinking of the powers of life, while others will recover after most frightful hæmorrhages. All these considerations must influence to a certain degree the conduct of the accoucheur, when summoned to a case of unavoidable uterine hæmorrhage.

This species of hæmorrhage may either come on suddenly and with violence, and it may not subside until delivery or death take place, or it may be more gradual, and its threatening may have been indicated by occasional discharges of blood during the latter stage of pregnancy.* Any such discharges therefore in the last three months must be most cautiously watched; and as soon as labour commences, the accoucheur should endeavour to ascertain exactly the position of the placenta. Unavoidable hæmorrhages are very generally increased, while accidental hæmorrhages are usually diminished during every labour-pain. On no pretext is the accoucheur ever to leave the bed-side of a patient, when he has discovered that the placenta is situated over or near to the os uteri. No premature interference however, even under these circumstances, is justifiable. He must cautiously wait until the

* It is a well-ascertained fact, that hæmorrhages occurring near the full period of pregnancy, are almost invariably more enfeebling and dangerous, than at any earlier period. If properly treated, women seldom die from hæmorrhage before the sixth month.

uterine orifice opens, and becomes sufficiently dilatable to permit the introduction of the hand without force or violence. Cases indeed of unusual difficulty will sometimes occur, where in consequence of the alarming loss of blood immediate delivery must be attempted, even although the os uteri is rigid and unyielding; but dire necessity only should induce him to hazard the consequences of such violence. In duty to himself, the accoucheur should acquaint the friends of the patient with the extreme danger of the case, previous to making any attempts to turn and deliver the child. The following directions cannot fail to be duly appreciated by every physician who may be summoned to the relief of a case of unavoidable hæmorrhage.

"As to whether the hand should be passed through the placenta, or at its edge, we must be guided by circumstances; always introducing it slowly and gently wherever we find the resistance or opposition least. If it have been well soaped previously, it will seldom cause acute pain, the parts being relaxed and lubricated by the discharge. It will much facilitate the operation to have the hips placed as near the edge of the bed as may be, the nurse-tender at the same time elevating, as much as she can, the right knee. When we have so far succeeded with the delivery as to have got the feet into the vagina, the remainder should be conducted slowly, in order to permit the mouth of the womb to yield gradually to the passage of the child's body and head; and also, to encourage a regular and perfect contraction of the uterus; which in such cases is of the last importance to the welfare of the patient. Too much care cannot be observed to prevent loss of blood after delivery; even a slight subsequent discharge is much to be dreaded." 95.

Before leaving the bedside, we must carefully examine the state of the uterus, and ascertain, even by manual exploration if necessary, that it has continued properly contracted; as inward hæmorrhage will sometimes take place, and, the cavity of the womb becoming distended with blood, the patient will expire, although no outward draining has been observed.

Twenty-four cases of profuse hæmorrhage previous to the birth of the child occurred during the Mastership of Dr. Collins at the Dublin Hospital. Eleven of these were of the unavoidable sort. In eight of the eleven cases, the presentation was natural; in four of these the children were turned; in one the labour was completed without assistance; in one the forceps; and in the other two the perforator was used. Of the remaining three, two were footling, and one was a breech case. Six in all of the children were born alive. Two of the women (and in these the children had been turned) died; all the rest recovered. In one of the fatal cases, immediate delivery was absolutely necessary, although the parts were not sufficiently relaxed, and the os uteri was but very imperfectly dilated. On dissection, "a laceration of the neck of the uterus anteriorly and to the right side was discovered, commencing at its junction with the vagina and extending upwards."

In the other fatal case, the death was owing to the excessive exhaustion alone: hæmorrhage had continued for some time after the birth of the child.

One of the cases where the patient recovered is extraordinary, from the placenta having been expelled many hours before the delivery of the child, which however was quite putrid. It is altogether so singular, that it deserves to be recorded in the words of the author.

"G. J. at her full time; was admitted in a state of extreme debility, her pulse so weak and frequent as not to be counted. The foot was found in the vagina, so putrid that the skin peeled off on the slightest touch. The discharge was fetid. Stimulants and cordials were freely given, and the child brought away without difficulty. The uterus remaining enlarged and relaxed, the hand was passed to remove the placenta; when there was *none* to be discovered; nor was there any hæmorrhage. The membranes had ruptured and the waters been discharged, a fortnight previous to admission, from which time, until the evening before she was brought to Hospital, she had more or less hæmorrhage. It was now ascertained that the placenta had been expelled the evening before her admission, and separated by the midwife in attendance. She had been twice visited by a medical practitioner, who *bled her* and gave *purgatives.* She left hospital well on the 13th day." 103.

*Accidental Uterine Hæmorrhage,* or that which is caused by a separation of the placenta from any part of the womb except its orifice, is next to be noticed. It is a much less alarming accident than the unavoidable hæmorrhage. In the majority of cases it will subside under appropriate treatment, without any direct interference with the delivery.

The first and leading duty of the accoucheur, is to ascertain whether the placenta is situated at or over the orifice of the womb: if it is not, he has reason to hope that, unless the hæmorrhage be unusually profuse, or some unforeseen accident occur, a prudent management may conduct the labour to a fortunate issue. To check hæmorrhage before the coming on of labour, a recumbent posture, perfect quiet, refrigerant drinks, opiates (occasionally) and cold applications to the external parts will always be judicious. If these means fail, rupturing the membranes so as to discharge part of the liquor amnii, and induce a certain degree of uterine contraction, is "almost invariably a safe and effectual expedient." This simple operation will often suffice; and we should therefore never proceed to turn and extract the child (a practice which has been too indiscriminately recommended in such cases by Dr. Burns) until we have watched the effects of merely discharging the waters. The caution so earnestly impressed by Dr. Collins in the following sentence should be well remembered by the young accoucheur.

"I know of no operation, more truly dangerous both to mother and child, than the artificial dilatation of the os uteri and turning the child; and confident I am, that the practitioner who adopts such a line of practice, *except from strict necessity,* will often have abundant cause to regret his proceedings." 108.

The experience of the most experienced accoucheurs of the present day demonstrates the very decided success which follows the rupturing of the membranes, when the placenta is not attached at the orifice, and the hæmorrhage is therefore of the accidental, and not of the unavoidable species. Dr. Merriman reports 30 cases, and Mr. Rigby 51 cases, of alarming accidental hæmorrhage successfully treated in this manner. The injudicious advice therefore of Dr. Burns, contained in the following extract from his work, cannot be too strongly deprecated.

"In those cases where the placenta presents, few practitioners would think of trusting to the evacuation of the liquor amnii; they would deliver. If then delivery be considered as safe and proper in one species of flooding, it cannot be dangerous in the other; and whenever interference in the way of operation is necessary, the security afforded by the introduction of the hand will much more than compensate for any additional pain. But even in this respect the two operations are little different, if properly performed." 108.

To rupture the bag of the membranes, the finger will generally suffice: if the os uteri however be very high up, or the membranes unusually lax, the use of a blunt probe may be necessary. When the hæmorrhage continues after the rupturing of the membranes, so as at all to endanger the life of the mother, we must then resort to immediate delivery, either by turning or with the forceps. If the child be dead, as may be ascertained by the stethoscope, "we should rather perforate the head and extract, than expose the mother to the danger of turning."

Very generally the child is still-born after any very profuse uterine hæmorrhage. The extreme importance of withdrawing the child gradually and uniformly, and of employing at the same time other means, as firm pressure over the abdominal tumor, so as to insure a complete and permanent contraction of the uterus, cannot be too frequently and too earnestly insisted upon. The life of the mother depends upon our care in this respect.

It may be necessary to administer wine, brandy, and other cordials before as well as during the attempts to deliver.

The following succint account of the appropriate treatment in unavoidable and in accidental uterine hæmorrhage may usefully close our remarks:

"Where the placenta is fixed *over or near the os uteri*, nothing but delivery will put a stop to the loss of blood; whereas, if the placenta *be not so situated*, the discharge not profuse, and the patient's strength not much reduced, we should use every effort to arrest it, by the means commonly recommended, and wait for nature's assistance in the expulsion of the child. If, however, the natural efforts be not likely to effect this in a reasonable time, and the strength be gradually failing, it will be necessary to rupture the membranes, and should this not succeed in checking the hæmorrhage, we must deliver by art, carefully bearing in mind the directions laid down for the successful accomplishment of our object." 112.

The hæmorrhage which is apt to occur between the delivery of the child and the expulsion of the placenta is invariably connected with retention of the placenta, and with an imperfect contraction of the uterus. Very often these unfortunate circumstances are attributable to the earlier stages of labour having been hurried or unnecessarily interfered with. Some females indeed appear to have an unusual predisposition to violent flooding with every child, and others to a tedious and most obstinate retention of the placenta.

Dr. Collins permits the placenta to remain, if no hæmorrhage supervene, for two hours after the delivery of the child without interference. We consider this to be rather an unnecessary delay; we have never witnessed any unpleasant effects from gently withdrawing the placenta, or rather from merely stimulating the internal surface of the uterus, by the hand introduced, to throw it off, within an hour after the child has been expelled.*

---

* This sentence, and indeed the whole of our review, as already stated, of Dr. Collins' work had been written before we saw Dr. Hamilton's observations. We had expected to have been able to have inserted it in our last number, but the more than ordinary press of matter has delayed it to the present one. The opinion expressed as to the propriety of removing the placenta within an hour after the expulsion of the child is sanctioned we are happy to find, by the high authority of the Edinburgh Professor. His words are: "From the earliest period of the author's professional life, he was taught to adopt two rules in

His advice also to the accoucheur "never to leave a patient for *at least one hour* after the placenta has come away, and she has been free from any unnatural discharge of blood" is surely not requisite when the labour has been regularly and uniformly natural.

Of sixty-four cases of hæmorrhage occurring between the birth of the child and the expulsion of the placenta during the mastership of Dr. Collins, seven proved fatal; two only however from the mere effects of the loss of blood. In six, the hæmorrhage continued for some time after the removal of the placenta; in 13 cases, the placenta was found firmly adherent to the uterus, and in 8 cases the hour-glass, or irregular contraction existed.

The very firm adherence of the placenta to the uterus, requiring its manual separation and removal, is always a most unpleasant complication; the uterus is apt to become inflamed in consequence, and this danger is increased ten-fold, if puerperal fever be prevalent at the time.

In one of the cases related by Dr. C. we observe that the adherent placenta had been allowed to remain for two hours, and in another case for three hours, although there had been occasionally slight hæmorrhages; we certainly should not have waited so long ourselves, before removing it. No advantage is gained by so tedious a delay, and the uterus is certainly less disposed to contract after an expiry of two hours than of one only after the expulsion of the fœtus. In removing the placenta when it has been firmly adherent, the accoucheur should be most watchful that the uterus contracts upon the placenta and the hand as they are gradually withdrawn.

Dr. Hamilton devotes a good many pages to the consideration of retention of the placenta from its morbid adhesion to the uterus, and from the hour-glass contraction of the womb. He alludes to the changes in structure which the placenta sometimes exhibits; as, its surface being covered with a film of osseous matter—bony spiculæ penetrating through its substance, or part of it being converted into a gristle, of a compact dense texture, and semi-transparent like melted horn. In detaching a morbidly adherent placenta Dr. H. deprecates the practice, commonly pursued, of insinuating the

---

respect to the management of the placenta, viz., to interfere upon the very first threatening of hæmorrhagy, and, where there were no untoward symptoms, to wait no longer than one hour for the natural efforts. Every year's experience has strongly impressed his mind with the utility of those precepts."

But we must enter our protest against one at least of the precepts contained in the following remarks on retention of the placenta occasioned by atony or imperfect contraction of the uterus: "The appropriate practice in this case is, to excite the contraction of the uterus. This is best done by the exhibition of a cordial; the application of heat to the lower part of the belly; and pressure upon the uterus by means of the hand.

If the contraction of the uterus be not secured by the employment of these means within an hour after the birth of the infant, a stimulant enema ought to be administered, and should that fail, the manual separation becomes indispensable." 280.

The application of heat to the lower part of the belly in such a case is surely most unwise and improper. We object also to the exhibition of a stimulant enema. It is only losing time and giving annoyance to the patient. All that is ever required is very firm pressure on the uterine tumor with the hand, and if this fail, the introduction of the hand into the vagina.

fingers between it and the surface of the uterus—a practice, he says, which is apt to excite inflammation of the womb, and sometimes even to produce laceration of its substance—and recommends that "pressure be made exclusively upon the fœtal surface of the placenta, bringing its circumference towards its centre, and detaching leisurely and carefully all that can be separated by this manipulation." Firm pressure on the uterine tumor from the abdomen must be steadily kept up all the time.

Dr. H. assures us that in his extensive experience he has very rarely observed any injurious consequences to arise from leaving the indurated maternal portion of the placenta in the uterus.

"He has attended many cases where two, three, or more days have intervened between the birth of the infant and the separation of the adherent indurated portion of the secundines, and he never witnessed any untoward symptom, such as flooding or subsequent irritative fever. In one case, a mass weighing eight ounces was retained five days without occasioning any symptoms indicating danger.

When it is thus necessary to leave a portion of the adhering mass, the progress of the case must be carefully watched, and particularly, the state of the lochial discharge must be examined at least twice a day, in order to ascertain when the sloughing of the retained portion is effected, for wherever that happens, a strong purgative enema must be administered." 299.

With respect to the symptoms and management of retention of the placenta in consequence of the hour-glass contraction of the uterus—an accident which if not speedily relieved is of most alarming danger—Dr. H. offers the following remarks. He is of opinion that its existence is indicated by the following characteristic symptoms: although uterine contractions follow the birth of the infant, there is no lengthening of the cord; the uterus examined through the abdominal parietes is felt like two rounded bodies joined together; and lastly when the cord is pulled gently, it readily comes down, but, on being let go, it recedes with a jerk. Dr. H. says that the hour-glass contraction of the uterus is "always the effect of mismanagement;" often of the improper protraction of the first stage of labour, as in the case of the Princess Charlotte; or of extracting the body of the child the moment the head is born, before the womb has properly contracted upon itself. The treatment to be pursued is thus laid down by Dr. Hamilton.

"For the purpose of remedying this cause of retention, an opiate enema should be first administered (provided there be no hæmorrhagy), and after half an hour, the practitioner should proceed to introduce his hand, guided by the cord, to overcome the constriction, to separate the placenta, and to secure the regular contraction of the uterus." 283.

Should hæmorrhage occur after the placenta has been expelled, firm pressure with the hand over the uterus, and the application of cold water will generally suffice; but if these means fail, then the most effectual are the re-introduction of the right hand into the uterus, and the firmly grasping of it outwardly with the left one at the same time, combined with the internal administration of stimulants (if the patient be very low) and the directing of a stream of cold water from a height on the hypogastrium.

A large opiate is usually necessary afterwards in such cases. Great restlessness occurring after any profuse loss of blood is always a dangerous symptom, and the sooner it is effectually checked by an opiate the better;

30 or 40 drops of laudanum in union with a cordial, such as brandy and water, may be given every twenty minutes, until the patient is lulled to quiet.

Dr. C. tells us that he has sometimes succeeded in allaying extreme restlessness by the injection of 30 or 40 drops of the acetum opii in cold wine and water into the rectum, where opium freely given by the mouth had failed.

Forty-three cases of hæmorrhage subsequent to the expulsion of the placenta occurred among the 16,414 labours. In one half of the cases it happened within the first fifteen minutes, and in the rest at various intervals, from half an hour to several days. In eight the hæmorrhage was internal; in five, both external and internal. Four of the women died; two only however from the effects of the loss of blood, and in both these cases portions of the placenta were found adherent to the uterus. We should be inclined to believe that most of the cases of profuse hæmorrhage after the expulsion of the placenta were owing to retention of part of the placenta. The entirely empty womb is much less subject to drainings of blood than when any foreign substance is within its cavity.

The eight cases of *internal hæmorrhage* reported by our author merit the attentive consideration of every obstetrical reader. Whenever a woman, soon after delivery, becomes faint, low, and pallid, without any obvious cause, the accoucheur ought forthwith to suspect the existence of this insidious accident. The introduction of the hand, the firm compression of the uterus, and the application of cold water in a stream to the hypogastrium are the most potent remedies.

On the subject of *puerperal convulsions*, we shall select only the most interesting and novel remarks of our author, as in our Number for last July we devoted several pages to the consideration of this most important disease.

Dr. C.'s experience agrees with that of Dr. Joseph Clarke, and of Dr. Merriman, that convulsions are much more common during first than during any subsequent labours, and (which is rather a curious circumstance), that in almost every case of convulsions, the presentation will be found to be natural. Dr. Denman has remarked, "that he thought for many years that convulsions only occurred where the head presented, but experience has now proved that they sometimes occur in preternatural presentations."

Of 30 cases in our author's practice, five proved fatal. In 15 of the 30 cases, the patients were delivered by the natural efforts, in six by the forceps, in eight by the perforator and crochet, and in one only the feet presented. In 18 cases the convulsions subsided after delivery, in 10 they continued for some time after, and in two the attack did not come on till after delivery. Fourteen only of the children were born alive.

The post mortem appearances discovered in four (dissection was not permitted in the other case) of the fatal cases did not throw any light on the nature or cause of the disease; once only an encephalic lesion was found; "a quantity of effused fluid and blood was observed under the pia mater of the posterior lobes of the brain;" in three cases, the vagina had been lacerated, and in one there had been inflammation of the peritoneum.

Dr. Denman long ago remarked the great liability of patients to abdominal inflammation, after attacks of puerperal convulsions. Other writers have confirmed the accuracy of the observation. In the treatment of puerperal convulsions, Dr. Collins trusts chiefly to large bleedings, and to the use of tartar-emetic, given in doses of from a quarter to half a grain with a few drops of laudanum, every twenty or thirty minutes, until complete nausea and general relaxation of the system are induced. Pounded ice, or iced water, should be applied to the head at the same time. However alarming and dangerous convulsions are, and important although it may be, in such cases, that delivery be speedily accomplished, Dr. C. deprecates the practice of artificial dilatation of the os uteri, for the purpose of turning and extracting the child, as recommended by Dr. Burns; "indeed," says he, "there are few instances under any circumstances (some hæmorrhages excepted) where I should recommend this practice; but in *convulsions* I consider it particularly injurious."

The tartar-emetic treatment will be found of the greatest utility in enabling the accoucheur to gain time, until the os uteri is duly dilated. We shall give the abridged particulars of one case to illustrate the practice recommended by Dr. Collins. A. B. was brought to the hospital in labour, having had several convulsive fits for some days previously; on admission, she was quite insensible, and was scarcely placed in bed, when a severe fit comes on. She bled to twenty ozs. and two table-spoonfuls of a mixture—composed of eight grains of tartar-emetic, forty drops of laudanum and eight ounces of water—were given every half-hour.

The fits returned in quick succession: the bleeding was twice repeated, ten grains of calomel were given, the antimonial mixture continued, and cold lotion applied to the head. Hitherto the os uteri was undilated; but in the course of an hour it began to expand, and gradually attained the size of a half-crown. During this time the patient had several fits, some of which were extremely violent; and before she was delivered on the following morning with the forceps, she had had in all *seventeen fits* since her admission into the hospital. In the intervals of the fits, the patient was incessantly tossing about, and seemed insensible to every thing. After the expulsion of the placenta, thirty drops of laudanum were given, and the cold applications kept steadily to the head.

During the following four hours she had seven fits, but none were very severe, and she remained in a stupid dozing state. The tartar emetic mixture was resumed; but as the unpleasant symptoms did not abate, twelve ounces of blood were drawn, and powerful purgatives were administered;—a blister also was put on the nape of the neck. In the course of twelve hours, these active remedies had induced a considerable abatement of the lethargy and general oppression. For several days the mind was always more or less disturbed, and it required repeated application of leeches to the temples, and the employment of the most rigid antiphlogistic treatment to lead the case to a favorable issue.

The details of this case shew that, even when the convulsions are frequent and severe, we may with perfect safety defer for some time any interference with the delivery. In many of the other examples recorded by Dr. C. the patients underwent several violent paroxysms, before the labour was completed either by natural or by artificial means. The young practitioner must not therefore allow himself to be alarmed into any rash attempts to extract the child, if the labour be not sufficiently advanced, and the parts are not duly dilated. A fair trial is to be given to the powerful remedies Dr. Collins so urgently recommends, before any manual or instrumental interference is resorted to.

With respect to convulsions occurring after the delivery of the child Dr. C. is of opinion that they are less frequently dangerous than those which precede it. His experience on this subject differs from that of Dr. Ramsbotham, who has stated in his practical observations.

"That convulsions, after delivery, are more untractable and prove more frequently fatal, than where they occur previous to, or during labour." He continues, "I have remarked that when they come on under either of the latter circumstances, and continue after delivery, whether it may have been effected naturally, or hastened by art, they generally prove destructive to the patient." 231.

The use of opium in puerperal convulsions has been a "vexata quæstio" among medical writers. Some, and these of high eminence, as Hamilton, Burns, and Ramsbotham, have unqualifiedly rejected and denounced it as highly dangerous, while Dr. Collins assures us that "ample experience, however, has convinced me, that it is not only harmless, but *highly beneficial*, in those cases where the fits *continue after delivery* ; and I should hope the cases adduced will prove satisfactorily, that it is also useful under many other circumstances, where proper steps had been previously taken. Its combination with Tartar Emetic, and occasionally with calomel, is most advantageous."

The dispute may we think be easily adjusted. Whenever convulsions are connected with plethora or increased activity of the circulation, opiates must be injurious; but when they appear to be the result of excessive irritability, or of exhausted energy, they will be found, if administered with judgment, and associated with other means, as cold to the head and blisters to the neck, of great service. All *unqualified* praise or condemnation of particular remedies, either in this or in any other disease, is unscientific, and indicates a prejudiced mind.

*Rupture of the Uterus or Vagina.* This most melancholy accident is apt to be induced, whenever the uterus during parturition is excited to very violent expulsive action, especially if there is at the same time any unusual obstruction to the delivery of the child. Hence, narrowing of the pelvis, unnatural rigidity of the soft parts, and a disproportionate size of the fœtus are, one or all of them, very generally present in cases of rupture of the uterus. The operation of turning is unfortunately not an unfrequent cause of this accident, and even the rash and violent efforts to remove an adherent placenta have induced it. Dr. Collins has known the mouth of the womb to be torn by the imprudent use of the forceps. He denounces in the most impressive language that overhasty and most dangerous practice of resorting to manual or instrumental interference, before the os uteri and other soft parts are duly dilated. We fear that too many accoucheurs do not weigh sufficiently the serious and even fatal consequences which sometimes follow the operation of turning, injudiciously attempted, or rashly executed. The symptoms of rupture of the uterus are usually most appallingly characteristic : the sudden cessation of the labour-pains, the feeling as if something had given way within, the recession of the child, the deadly faintness, the acute crampish pain in the region of the stomach, and the urgent distress on pressure over the uterus, too surely proclaim the nature of the accident. Sometimes, however, when the extent of the injury is not great, the symptoms are more obscure, and even the uterine action may continue vigorous enough to assist in the delivery of the child. Under such circumstances, the accoucheur may remain in total ignorance of the accident, until it is ascertained either on the removal of the placenta, or after the death of the patient. The part where the rupture usually commences, is at the junction of the cervix uteri with the vagina, either anteriorly or posteriorly. No instance of rupture of the fundus has been witnessed at the Dublin Hospital, and we do not recollect any on record. In nine out of thirty-four cases, the peritoneal coat of the uterus was uninjured, although the muscular substance of the cervix was extensively ruptured; in one case, which had been attended with all the symptoms of the accident, no rupture could be discovered on dissection, and the only lesion was the existence of numerous lacerations of the peritoneal membrane, on the posterior surface of the uterus.

The treatment which may be required of the medical attendant, must depend in some degree on the circumstances of the case. The paramount object is to effect the delivery of the child as speedily as possible, without adding to the danger, already great, of the mother. In some rare cases, the labour being far advanced and the parts well dilated, the child is expelled without assistance, and alive. This happened four times out of thirty-four cases at the Dublin Hospital. Some writers have far too indiscriminately recommended the application of the forceps in cases of ruptured uterus, with the praiseworthy motive of saving the life of the child; but this is a fallacy. From the experience of Dr. Collins and other experienced accoucheurs, it appears that "the child dies shortly after the rupture takes place." Dr. Burns indeed has said, that "the child sometimes lives for hours;" but he has not given any case in point. If we therefore have reason to be satisfied that the child is dead, much time may be lost by employing the forceps, unless the head be so far advanced that it can be readily grasped, and the child quickly extracted. In three of the thirty-four cases which occurred in Dr. C.'s practice, attempts were made to deliver with the forceps, but without success. The perforator and crotchet will be found to be, in a large majority of instances, preferable to the forceps. The uterus must be kept by an assistant as steady and fixed as possible, and the application of the instruments is to be made with great caution and gentleness; else the fœtus may recede, or even escape into the abdominal cavity by the pressure employed. When this last-named accident has taken place, the accoucheur is not, as was wont to be the case, to desert his patient and leave her unassisted. Although the prognosis is most unfavourable, he is still to make an attempt to deliver by introducing the hand through the rupture into the cavity of the abdomen, and bringing down the feet without delay; the placenta is then to be withdrawn, and any portion of intestine, which has become entangled between the lacerated edges, should be cautiously replaced.

Of the 34 cases, two only recovered. In one of these, the arm and shoulder presenting, delivery was effected by perforating the thorax and by bringing down the breech; and in the other, the head being low down, the perforator was applied directly to it, and the extraction completed with the crotchet: the rupture was most extensive, and through it a portion of intestines had escaped. Both patients were discharged nearly well in about four weeks after the date of the accident. The latter patient has been twice pregnant since; and both times she has given birth to living children, with the first of which premature labour was induced. The fatal event took place at various intervals after delivery: sometimes immediately afterwards, in other cases not for many hours, or even many days. The latest period mentioned is the 25th day. Two of the children were born alive: four were delivered by the natural efforts; 19 by the crotchet; 7 were extracted by the feet; in two cases, the delivery was effected by lessening the thorax, and bringing down the breech, and in the remaining two, the mode of delivery is not stated.

Dr. Collins has detailed seriatim the history of each of the 34 cases, and described the post-mortem appearances in many of them. The particulars cannot fail to be extremely interesting to the accoucheur, and the information communicated by so experienced and talented an adviser as Dr. C., must give a degree of self-confidence to the young practitioner, when called upon to act under the trying circumstances of rupture of the uterus.

Before quitting this subject, we may briefly allude to a well-authenticated case of that physiological curiosity, " the crying of the child in utero," related by our author. His testimony will be deemed unexceptionable.

" The most extraordinary occurrence in this case was the *respiration* and *crying* of the *child in utero;* both of which were heard, as distinctly as possible, four hours before delivery, the latter at a distance of some yards from the couch on which the patient was lying. These facts were witnessed by myself and assistants, besides several of the pupils, both by stethoscopic examination and otherwise. The head was, at this time, high in the pelvis; the soft parts partially dilated, and the waters but a short time discharged.

The cry was so distinct, that I imagined the child was placed merely under the bed-clothes. When called to witness this truly singular phenomenon, I little credited the truth of what I was told, and confess, had I not been present, I should have remained sceptical.

How forcibly should this fact prove the uncertainty of some of the tests most confided in, as indicative of the murder of new-born infants! It also affords the medical witness in such cases, a salutary caution, in addition to those so ably advanced by the learned Doctor William Hunter on this subject."* 299.

*Prolapsus of the Umbilical Cord.* The practice of the English and French accoucheurs is strikingly contrasted in their treatment of this accident. We need scarcely mention that the prolonged compression of the umbilical cord in consequence of its premature protrusion, and the consequent interruption to the fœtal circulation, is inevitably fatal to the child. The event of the case will depend on the delay of the delivery. The average mortality among the children, in the cases of prolapsed cord at the Dublin Hospital, is three-

* In a recent No. of the Bulletin Med. de Bourdeaux, a Dr. Barsac relates a very extraordinary case. The crying of the child was heard, we are told, for three days successively, at intervals, in the eighth month of pregnancy, long before labour commenced. The particulars of the report are however not satisfactory.—*Rev.*

fourths of the whole (73 out of 97); whereas, in the Maternité in Paris, it is greatly inferior. This difference is attributable altogether to the very different modes of treatment pursued. In France, the operation of turning is resorted to, whenever there is any great difficulty in the delivery, or any danger incurred by the child—the safety of the mother being often sacrificed for the attempted salvation of her offspring. In this country, we are more chary of the parent's life, and therefore more prodigal, it may be said, of that of the child. None of the mothers in the 97 cases sustained any injury in their labours. It has been very generally remarked that the mortality among the inmates of the Maternité at Paris, is greater than in any obstetrical institution in this country, the proportion of deaths being nearly one in every 24 patients, and, during some years, one in every 12 or 13. "In *one* year (says Dr. C.) a greater number of deaths, by nearly *a half* occurred, than were met with during my *seven years'* residence in the Dublin Lying-in Hospital, where 16,414 women were delivered. Baudelocque reports 700 deaths in 17,308 deliveries; whereas in 16,414 we had 164."

In judging therefore of the propriety of any practice in midwifery by the number of deaths among the children born, we ought to be informed, at the same time, of the mortality among the women. This omission Madame Boivin and other French authors are too often guilty of. Delivery with the forceps, or by turning the child, is resorted to in the French hospitals in almost every case of prolapsed cord. To the forceps, provided always the circumstances of the case and the stage of the labour be favourable to its employment, there can be no objection; but as for the operation of turning, Dr. C. does not hesitate to assert that "the risk to the mother is, in the majority of cases, so great as to forbid its employment; nor do I think the practitioner justified by the circumstances (of the cord being prolapsed), in so greatly hazarding his patient's life."

Such being the difference of opinion among French and British physicians as to the management of prolapsus of the umbilical cord during labour, we cannot be surprised at the difference in the mortality of the children so born.

Before resorting to any manual or instrumental assistance, it is the duty of the accoucheur to try the simpler method of pushing up the funis through the os uteri with the fingers, and of endeavouring to keep it secured above the head by means of a piece of sponge introduced. Unfortunately, in most cases, the cord is again protruded as soon as the fingers are withdrawn. Dr. C. suggests the following method.

"We tried, in a few instances, to return the funis completely into the uterus; by passing a piece of soft twine through a long gum-elastic male catheter, so as to retain the funis exactly at its extremity; and, when thus fixed, a strong copper wire was introduced into the catheter, in order to render it sufficiently firm to carry up the cord; when elevated as far as practicable, by these means, the wire was *withdrawn*, and the catheter retained in its position during the subsequent expulsion of the child. With this contrivance there was not a sufficient number of trials, as it was but a short time previous to my retiring from the Hospital that this expedient was had recourse to. Doctor Evory Kennedy, the present master, will, I am sure, lose no opportunity of testing its utility." 346.

When it is ascertained that the child is dead, how is the accoucheur to act? Is he to allow the labour to be completed by the natural efforts, or ought he to expedite it, if not far advanced, by perforating the head, and bringing it down with the crotchet? Drs. Denman, Burns, and others, have advised the case to be left to Nature, unless an unusual or dangerous delay occurs; an advice from which Dr. Collins dissents, on the ground that "he cannot see any advantage whatever, in permitting the patient to endure the pain and distress of labour for hour after hour, which must be accompanied with some risk, when, by lessening the head, we can, without the least injury, terminate the labour." He reports twelve cases, where this practice was adopted with great advantage to the patients.

Much yet remains of this valuable work to review. Our limits permit us to do little more than glance only at the other contents. In the interesting chapter on twin births, the author alludes to the well-established but often forgotten fact, that the mortality among women after twin-labours, is considerably greater than after single-birth labours (nearly as 5 to 1), and he inculcates the necessity of more than ordinary attention to the after-treatment, seeing that all casualties, whether convulsions, hæmorrhage, fever, &c. are more dangerous under the former than under the latter circumstances.

The presence of twins has been often detected long before the commencement of labour, at the Dublin Hospital, by the stethoscope, from the sounds of the two fœtal hearts being heard at different times and of different intensities.

In the management of twin-labours, Dr. C. recommends that, if the membranes of the second child remain unbroken, for half an hour after the expulsion of the first child (within this period the second delivery usually takes place), they should be punctured, and a dose of ergot of rye may be administered at the same time, with the view of exciting uterine action. If this be still tardy, and delivery is not accomplished within two hours or so after the expulsion of the first child, we should extract either with the forceps or by turning, as it has been very generally remarked that the second child is likely to be still-born, if left longer than two or three hours unassisted. The placenta of the first child is sometimes expelled in perfect safety, before the delivery of the second child.

There were four cases of triplets in the Dublin Hospital during the mastership of our author. All the women recovered, but more than one-half of the children died within two or three days after delivery.

The chapter on *puerperal fever* is occupied chiefly with the reports of cases, some fortunate and others fatal. These cannot but be interesting and instructive to the obstetrical physician. The morbid anatomy is not given with such minuteness and accuracy as we could have wished. No explicit reference is made in any dissection to the state of the uterine veins or lymphatics, which, (as has been shewn by the researches of Drs. Lee, Tonnellé, Dance, Velpeau, and others,) are so very generally diseased. In every case examined, there was effusion into the abdomen. This effused fluid was either a straw-coloured serum, a sero-purulent matter, or "a bloody serum, with quite a glutinous fluid when rubbed between the finger and thumb." The surface of the intestines and of the uterus was usually coated with layers of lymph, giving rise to adhesions of greater or less tenacity. The peritoneum was invariably, in parts at least, highly vascular and injected.

"The uterus in the great majority was quite natural in appearance; in some it was soft and flabby, and in a few unhealthy matter was found in its sinuses. The ovaries, in numerous instances, had suffered much in structure from the effects of inflammation; being generally much enlarged, and so softened in most as to be broken in pieces by the least pressure."* 398.

The treatment generally pursued by Dr. C. consisted in the repeated application of large numbers of leeches, followed by hot fomentations to the abdomen, in the liberal use of calomel and opium, and in frictions with the mercurial ointment. Venæsection and blisters were occasionally, but not frequently resorted to. When a purgative was required, equal parts of castor-oil and spirit of turpentine were found to be the most useful. Dr. C. alludes, in more than one place, to the extreme danger of unnecessary manual interference with labours, and especially of the introduction of the hand into the uterus, while puerperal fever is prevalent.

"We had several severe attacks (says he) where we were compelled so to interfere, in consequence of profuse flooding or retention of the placenta; yet we were from experience so fully aware of the danger so long as puerperal fever prevailed in the Hospital, it was with the greatest possible reluctance that we had recourse in any case to this expedient." 133.

With some observations on still-born children, and on children dying in the hospital soon after birth, this admirable volume, which has afforded us such copious materials for analysis, is brought to a close. We cannot too highly recommend it to our readers; to the younger practitioner it will be found quite invaluable, as a clinical commentary on the more systematic treatises on midwifery, and the oldest and most experienced may rise from its perusal an abler and a wiser man. The two volumes, entitled "Practical Observations on Midwifery," published a few years ago by Dr. Ramsbotham, senior, have a similar scope and practical tendency to the present one of Dr. Collins. A diligent study of these two excellent works may save the accoucheur many an hour of embarrassment and distress, and perhaps, too, many a long year of unavailing regret for errors which, when once committed, can never be retrieved.

---

A Treatise on the Chemical, Medicinal, and Physiological Properties of Creosote, illustrated by Experiments, &c. By *John Rose Cormack.* Octavo, pp. 154. Edinburgh and London, April, 1836.

This is a very creditable essay of Mr. Cormack's, and we augur well of its author. The work is divided into two parts and five chapters—in each of which Mr. C. evinces acumen, science, and research. Creosote is now at-

---

* "I coincide in opinion with those who consider that the change of structure observed in the ovaries of women who die of puerperal fever, accounts satisfactorily for the sterility not unfrequently observed in young females who recover from this disease."

